# Neurofeedback As a Treatment for Major Depressive Disorder – A Pilot Study

**DOI:** 10.1371/journal.pone.0091837

**Published:** 2014-03-18

**Authors:** Frenk Peeters, Mare Oehlen, Jacco Ronner, Jim van Os, Richel Lousberg

**Affiliations:** 1 Department of Psychiatry and Psychology, South Limburg Mental Health Research and Teaching Network, EURON, Maastricht University, Maastricht, The Netherlands; 2 Department of Instrumentation, Faculty of Psychology and Neuroscience, Maastricht University, Maastricht, The Netherlands; 3 King's College London, King's Health Partners, Department of Psychosis Studies, Institute of Psychiatry, London, United Kingdom; VU University Medical Center, Netherlands

## Abstract

**Background:**

There is growing interest in neurofeedback as a treatment for major depressive disorder. Reduction of asymmetry of alpha-activity between left and right prefrontal areas with neurofeedback has been postulated as effective in earlier studies. Unfortunately, methodological shortcomings limit conclusions that can be drawn from these studies. In a pilot-study, we investigated the effectiveness of reduction of asymmetry of alpha-activity with neurofeedback in depressed participants with the use of a stringent methodological approach.

**Methods:**

Nine participants meeting DSM-IV criteria for major depressive disorder were treated with a maximum of 30 neurofeedback-sessions, aimed at reducing asymmetry of alpha-activity, over a 10-week period. No changes in the use of antidepressants were allowed 6 weeks before and during the intervention. Changes in depressive symptomatology were assessed with the Quick Inventory of Depressive Symptoms, self-report version.

**Results:**

We observed response in 1 and remission in 4 out of a total of 9 participants. The effectiveness appeared largest in female participants. The mean asymmetry of alpha-activity decreased significantly over sessions in a quadratic fashion. This decrease was associated with clinical response.

**Conclusions:**

This pilot study suggests that neurofeedback aimed at a reduction of frontal asymmetry of alpha-activity may be effective as a treatment for depression. However, this was an open label pilot study. Non-specific effects of the procedure and/or a beneficial natural course may have confounded the results. Randomized controlled trials will have to establish the efficacy of neurofeedback for depression.

**Trial Registration:**

Nederlands Trial Register NTR1629

## Introduction

There is growing interest in neurofeedback (NF) as a treatment for a variety of mental disorders including ADHD, anxiety, and depression [1], [2]. It is postulated that this technique, within an operant conditioning framework, helps individuals to regulate cortical electroencephalographic (EEG) activity while receiving feedback from a visual or acoustic signal. The resulting change in EEG activity is associated with a change in underlying cortical activation, and subsequently to result in a reduction of associated symptoms [3], [4].

On an electrophysiological level, major depressive disorder (MDD) appears to be associated with relatively more left than right resting (alpha, 8–13 Hz) activity in prefrontal regions [5]–[8], although some inconclusive studies exist (e.g., [9]–[11]. This difference in alpha activity between both prefrontal regions, has become known as alpha-asymmetry (AA) in MDD. To avoid confusion, it should be underlined that *increased* alpha activity in cortical structures is indicative of *decreased* cortical activation in those areas. AA is thought to represent reduced approach-related behaviours and reduced sensitivity to rewards in MDD [12]. Current research is equivocal as to whether AA in MDD represents a state-marker, an endophenotype related to risk for MDD, or both [8], [13]–[19]. As extensively discussed by Thibodeau and colleagues [15], methodological issues, sample-specific factors like gender distribution, and publication bias may explain inconsistencies in the current literature.

Notwithstanding these uncertainties, the premise that an episode of current MDD is associated with AA has been the starting point for AA manipulation with the application of NF as a treatment for MDD.

To date, case studies [1], [20]–[22], and a small randomized open trial [23] indicate that the increase of right relatively to left alpha activity at F3-F4 with the use of neurofeedback (alpha-asymmetry protocol) may be associated with a reduction in depressive symptomatology. This previous work has several limitations. First, NF treatment in the case studies was combined with psychotherapeutical sessions and lacked the use of state-of-the-art clinical instruments to assess psychiatric diagnoses and clinical change. The study by Choi et al [23], delivered only a total of 10 NF sessions during 5 weeks, which is considerably lower and less frequent than typically offered in the case studies and by NF practioners. Additionally, their participants suffered from subclinical levels of depression severity. Although the authors concluded that significant clinical change occurred in their active treatment group, clear criteria were not defined.

We decided to carry out a pilot study to address several questions. First, we aimed to examine whether NF is effective in the treatment of moderate severe MDD using current clinical instruments based on clearly defined response and remission criteria. Second, we investigated whether AA indeed decreased during the course of the NF sessions. Third, we examined the association between changes in clinical state and changes in AA. Lastly, the optimal duration of a single NF session for depressed subjects is unknown. Given fatigue and difficulties in concentration in MDD, we investigated the time-course of changes in AA during NF sessions to assess optimal session duration.

## Methods

### Ethics Statement

The protocol for this trial and supporting TREND checklist are available as supporting information; see Checklist S1 and Protocol S1. The Ethics Committee of Maastricht University approved the study. All participants provided written informed consent. The trial was registered by the Nederlands Trial Register, trial number NTR1629.

### Participants

**Figure 1:**
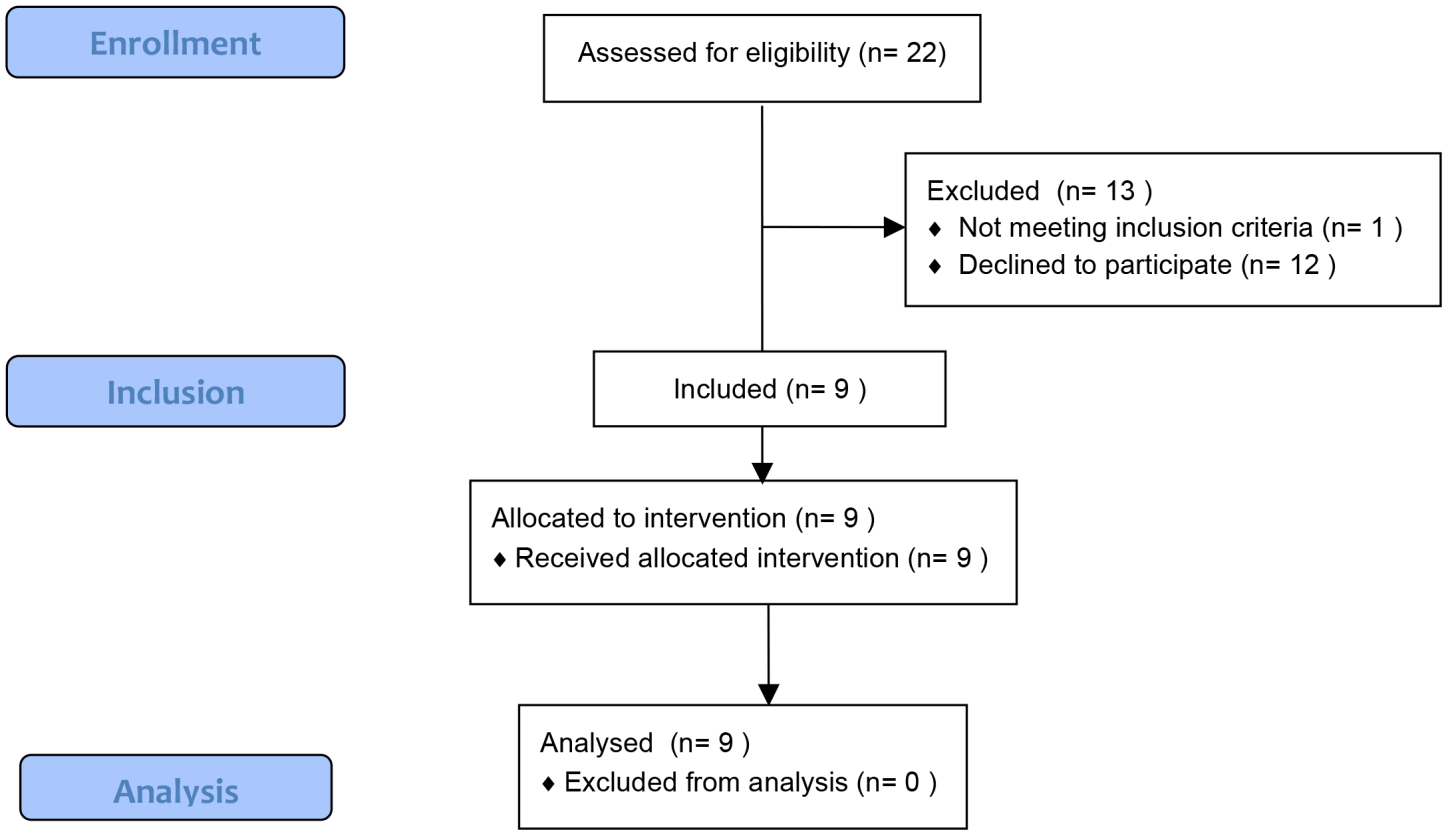
CONSORT flow chart .

The study sample consisted of self-referred treatment-seeking patients presenting at the mood disorders treatment program of an outpatient mental health care center in Maastricht, the Netherlands. While on a waiting list for treatment, patients were asked if they could be contacted by telephone by the first author about possible participation in the study. Recruitment started in March 2010 and ended in April 2012. Figure 1 shows the flow of participants through the study.

The inclusion criteria were a primary diagnosis of non-chronic MDD as assessed with the Structured Clinical Interview for DSM-IV Axis I (SCID-I; First, Spitzer, Gibbon & Williams, 1995) and right-handedness. Trained mental health care professionals conducted the SCID-I assessments. Exclusion criteria were 1) a primary axis-1 diagnosis other than MDD, 2) chronic MDD, 3) current or past brain lesions, 4) use of antipsychotics, mood stabilisers or benzodiazepines, 5) pregnancy, 6) elevated acute suicide risk and 7) insufficient command of the Dutch language. The use of anti-depressants was permitted when type and dosage were not changed 6 weeks before and during the study. Co-morbid axis I diagnoses were allowed.

### Instruments

The severity of MDD was measured with the 16-item Quick Inventory of Depressive Symptoms self-report version [24](QIDS- SR_16_). The QIDS- SR_16_ is a self-report questionnaire consisting of 16-items resulting in a total score between 0 and 27. Participants filled out the QIDS- SR_16_ prior to each NF-session. We defined response as a reduction of at least 50% on the QIDS- SR_16_ score, and remission as a QIDS- SR_16_score of ≤6 (www.ids-qids.org).

### Procedure, EEG recording and quantification

All EEG recordings took place in an electrically shielded room. While participants were seated in a chair, Ag/AgCl electrodes were placed on Fz, F3, F4, Cz, C3, C4, T3, T4, Pz, P3 and P4, Oz, O1 en O2 using the international 10–20 system [25]. The central, parietal, temporal, occipital and eye signal activity were only measured at the first and last NF-session. During the other sessions, electrodes were placed only on F3 and F4. To control for possible vertical eye movements, an electro-oculogram (EOG) electrode was placed 1 cm under the midline of the left eye. EEG electrodes were referenced with averaged earlobes (A1 and A2). A ground electrode was placed at the forehead. In order to reduce skin resistance, Nuprep scrub gel was used. All electrodes were fixed using 10–20 conductive paste. Impedances were kept below 5 kΩ.

Data collection was channelled through an acquisition PC with a BrainAmp DC EEG amplifier (Brain Products) using a 1000 Hz sample frequency. Online calculations were done by a filter written for BrainVision RecView. The data was epoched online into 2.048-s epochs that overlapped by 75% and then transformed by a fast Fourier transform (FFT) to the frequency domain (frequency resolution 0.488 Hz). Every 0.512 second, the power within the alpha frequency band (7.8 Hz–13.1 Hz) of both F3 and F4 was calculated. AA was computed as the difference of the natural log-transformed F3 and F4-alpha power: Ln(F3-alpha) – Ln(F4-Alpha). Present asymmetry was subsequently compared to the personal mean baseline asymmetry. The result of the calculation was sent to a stimulus PC running Presentation stimulus delivery software (Neurobehavioral Systems) with an 8-bit parallel port (LPT-port) to control a paradigm showing a visual representation of the asymmetry. In the Presentation paradigm, the last 20 values of the asymmetry are used in a moving average to prevent ‘jitter’ in the feedback. Participants received feedback with visual feedback; they were instructed to increase the level of a thermometer that was shown on a flatscreen. Additionally, a numerical score below the thermometer indicated their actual total performance. This score was adjusted (i.e. increased) continuously by a number ranging from 0 and 128, depending on the level of the thermometer. In this way a good actual performance (a shift in asymmetry in the desired direction) resulted in an increasing total score. A big shift in the desired direction resulted in a rapidly increasing total score, whereas a small shift in the desired direction resulted in a slow increasing total score. A shift in the undesired direction produced no change in total score. The purpose of this total performance score was to give participants feedback on the differential effect of the sessions.

Upon arrival in our laboratory, subjects were shown the facility and the monitor that displays the thermometer and the numerical score. After we had established a good EEG signal, they were just instructed to try to increase the level of the thermometer by trial and error. A typical response of subjects was the question if we could provide any detailed instructions that could be helpful in increasing the thermometer level. We outlined that there are no specific strategies (relaxation, thinking about positive experiences in the past or imagining experiences like being on a sunny tropical island) known to be helpful in this respect based on our experience; we had tested this with healthy volunteers (unpublished data). Moreover, there is concurrent empirical work showing that specific strategies used by participants are not beneficial in improving the magnitude of the effect of NF in targeted brains areas [26], [27]

Participants were treated with a maximum of 30 NF sessions that each consisted of 3 feedback-blocks of 8 minutes duration. These were separated by two blocks of 5 minutes rest. Block-duration was based on information in previous reports and information from NF-practitioners. Sessions took place 3 times a week. Before and after each session baseline activity was measured during 5 and 3 minutes respectively. If no clinical improvement was observed after 30 sessions, the participants were offered standard evidence-based treatment for MDD.

### Statistical Analysis

All statistical analyses were performed with SPSS version 20. Every block during the NF-sessions (baseline, feedback-block 1 to 3, and a post-NF baseline) was cut into 0.5 second segments: baseline was cut into 565 segments, feedback-block 1 to 3 into 916, and post-NF baseline into 331 segments. For each segment, AA was computed as the difference of the natural log-transformed F3 and F4-alpha power: Ln (F3-alpha) – Ln (F4-Alpha). All segments with eye blink activity (EOG > = 50 microVolt) were removed from the dataset.

Electrophysiological data have a hierarchical structure. Thus, multiple EEG observations (level 1) are clustered within participants (level 2). Multilevel analyses take the variability associated with each level of nesting into account [28]. Multilevel linear regression analyses were applied to the EEG data. The difference between conditions was analysed by use of multilevel regression with the dummy variables for condition as the independent predictor variables and AA as the dependent variable.

To examine linear and non-linear decreases in AA during each session, a multilevel regression analysis, with random intercept and random slopes, was conducted using time and its quadratic term as independent variables and AA as dependent variable. The optimum for every feedback condition was determined by calculating the top of the fitted parabola. Linear regression was used to assess the change in QIDS- SR_16_ scores during the course of the NF sessions.

The relation between QIDS- SR_16_ and AA over time was analysed by use of multilevel regression, using AA as dependent variable and QIDS- SR_16_, session and their interaction term as independent variables.

## Results

### Clinical outcome

Nine participants, 4 female, were included in this pilot-study. Mean age was 46.6 years (S.D. 11.7); mean baseline QIDS- SR_16_ score was 18.4 (S.D. 7.2), indicative of moderate to severe MDD. Female participants were significantly less depressed than males both before (QIDS_female_  = 12.5; QIDS_male_  = 23.2; 95%-CI: 3.6–17.9; p = .01) and after (QIDS_female_  = 5.25; QIDS_male_  = 19.2; 95%-CI: 6.7–21.2; p = .004) treatment with NF. Table 1 shows demographic and clinical information about the individual participants. Five participants (4 female) met the criteria for response and/or remission after treatment. During treatment, mean QIDS- SR_16_ scores decreased significantly (B = −.15, p = .048, 95%-CI: −0.30– −0.002).

**Table 1:** Demograpic and clinical characteristics of participants.

Participant number	Gender	Age	Pre-treatment QIDS- SR_16_	Post-treatment QIDS- SR_16_	QIDS-SR_16_ change	response	remission	No. of NF sessions
1	Male	35	19	27	+8	no	no	29
2	Female	27	12	6	−6	yes	yes	24
3	Male	58	27	11	−16	yes	no	30
4	Male	52	30	19	−11	no	no	29
5	Female	41	9	6	−3	no	yes	30
6	Male	49	16	17	+1	no	no	15
7	Male	39	24	22	−2	no	no	24
8	Female	56	13	2	−11	yes	yes	30
9	Female	62	16	7	−9	yes	yes	30

Note. QIDS- SR_16_  =  Quick Inventory of Depressive Symptoms self-report version [24].

The experiences from the participants were mixed depending on their ability to increase the level of the thermometer. Most of them engaged in a competitive effort to surpass results from previous NF-sessions by striving for a higher numerical score (indicative of their actual total performance during the current session) that was displayed below the thermometer. Participants reported feelings of competence and pride alternated with frustration depending on their performance. Besides these affective responses, no sudden gains in mood were reported during or directly after NF sessions. The participants did not report any additional specific thoughts or feelings during the sessions that could be related to their performance during the session. Although not formally assessed with any questionnaire, participants did not spontaneously report any side-effects during NF treatment.

As can be seen in Table 1, the number of NF sessions varied between participants. Two subjects (numbers 6 and 7) withdrew participation due to a lack of any clinical effect, one subject (number 2) terminated the treatment upon achieving full remission.

### Electrophysiological outcome

**Figure 2:**
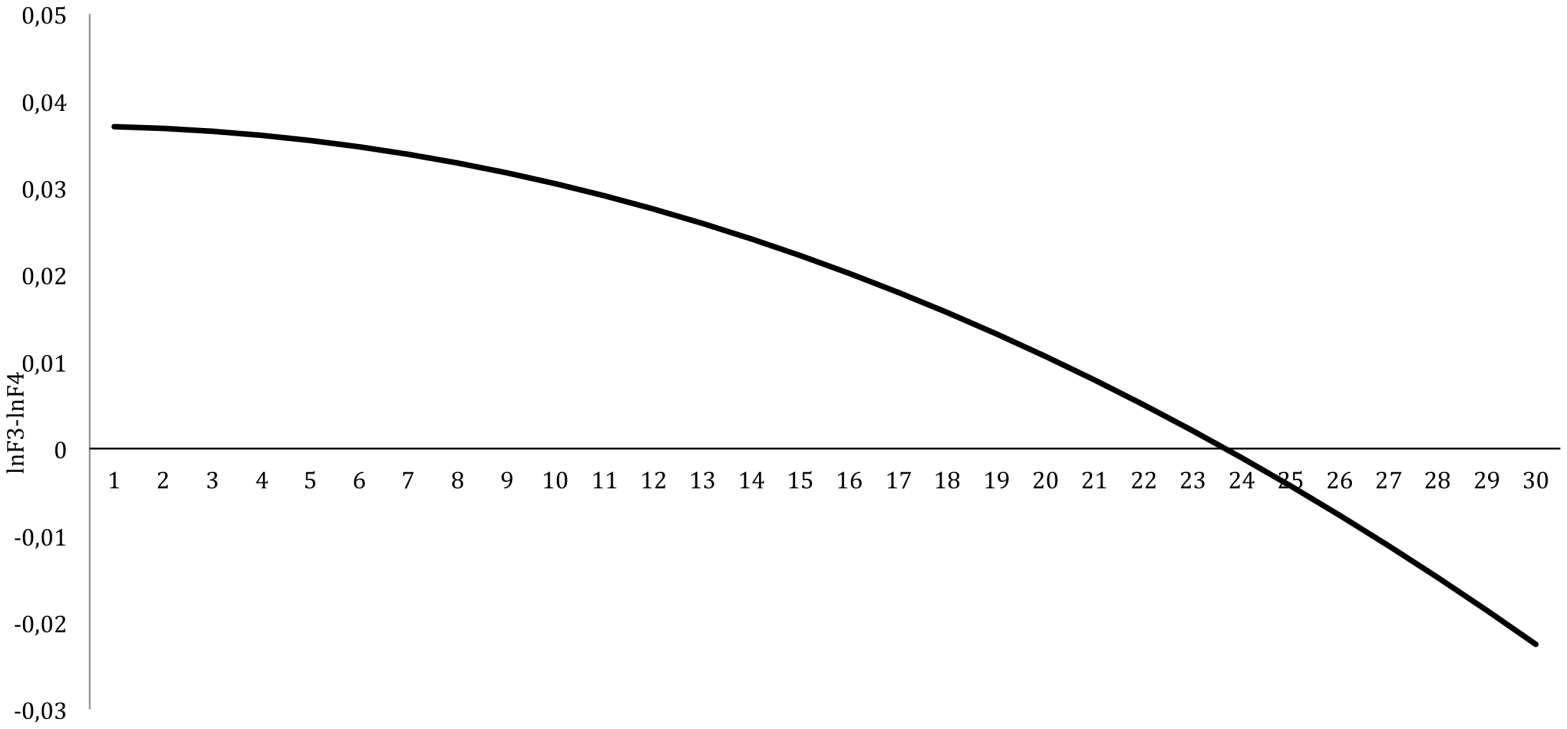
Change in mean baseline frontal alpha-asymmetry during NF treatment.

The mean baseline AA decreased significantly over sessions in a quadratic fashion (B = −.00007, t = −2.64, p = .008, 95%-CI: −0.00012– −0.00002). This decrease is graphically displayed in Figure 2. With respect to the random effects, both the intercept as the time effect were significant (p<.05). The intra-class correlation of this model was 0.012. QIDS- SR_16_ scores were significantly correlated with AA at baseline (r = .008, p = .004). QIDS- SR_16_ scores were significantly correlated with AA at baseline (r = .008, p = .004). We also examined whether decreases in mean QIDS- SR_16_ scores were associated with a decrease in mean AA. This association was significant (B = −0.0002, t = −8.15, p<.001, 95%-CI: −0.0002– −0.0001).

**Figure 3:**
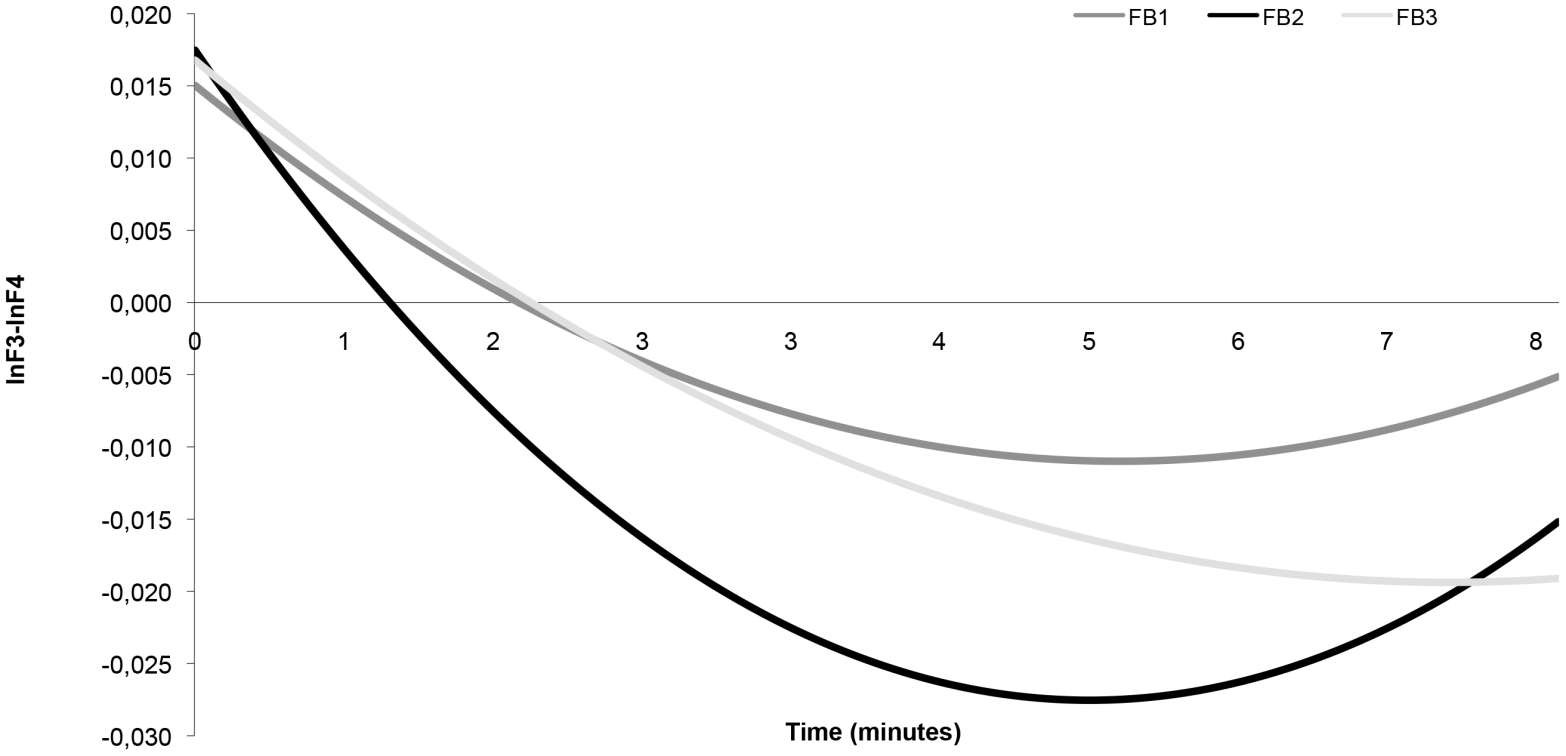
Time-course of the change in AA during the blocks during the NF sessions.

Next, we examined changes in AA during the 3 consecutive feedback-blocks (8 minutes each) within each NF session. The decrease of AA within the three 8-minutes blocks showed significant linear and quadratic effects (see Table 2). It should be noted that the beta's in Table 2 are small as a result of the logtransformation. Given the uncertainty about optimal length of NF-blocks, we also assessed the time-course of the change in AA during the blocks. The decrease of AA stopped after 5.18 (feedback block 1), 5.01 (feedback block 2), and 7.02 (feedback block 3) minutes respectively. This is graphically displayed in Figure 3. The optimal duration of NF-sessions is apparently shorter than the 8 minutes that we had chosen based on previous reports.

**Table 2:** Alpha asymmetry during the NF sessions.

		β	S.E.	t
Baseline	Intercept	.018	.022	.85
	Time	.000	.000	1.10
	Time^2^	.000	.000	−1.51
FB1	Intercept	.015	.037	.41
	Time	−.000	.000	−4.99***
	Time^2^	.000	.000	3.80***
FB2	Intercept	.018	.043	.41
	Time	−.000	.000	−9.11***
	Time^2^	.000	.000	7.17***
FB3	Intercept	.017	.042	.40
	Time	−.000	.000	−5.18***
	Time^2^	.000	.000	2.91**
Rest	Intercept	.035	.022	1.60
	Time	−.000	.000	−1.37
	Time^2^	.000	.000	.84

**p<.05, ***p<.001.

## Discussion

This NF pilot study into the effectiveness of NF as a treatment for MDD showed response in 1 and remission in 4 out of a total of 9 participants. This clinical change is in line with those from previous reports showing that neurofeedback, aimed at reducing AA, may be associated with a reduction in depressive symptomatology [1], [20]–[23]. We extended these studies by examining, i) a sample with moderately severe depressive symptomatology, ii) with the use of a structured clinical interview, iii) a robust instrument to measure clinical change with clearly defined outcome criteria, iv) a high number of NF sessions reflecting normal NF practice, and v) without additional therapeutic interventions.

During the treatment course with NF, resting-state AA significantly decreased and was associated with a reduction of depressive symptomatology. In the literature, there is uncertainty whether AA is a state-marker for MDD or should be considered a stable endofenotype for MDD [8], [13]–[19]. The current results provide support for the view that AA is a state-marker. Our methodology and statistical analysis may explain differences with some of the existing literature. In contrast to previous studies that tested changes in AA only before and after treatment (e.g., [17]–[19], we analyzed changes in AA continuously during the NF sessions, and analyzed within-persons changes with multilevel analyses. Such strategy makes it possible to analyse datasets with missing values (in this case due to invalid segments due to large EOG activity) and increases statistical power even when a small sample like ours is examined. It could be argued that the decrease in AA was small and reached significance due to high statistical power. However, there is no literature that indicates which magnitude of changes in resting-state AA should be considered as meaningful in an electrophysiological and biobehavorial respect. Future work will have to provide more information about this issue.

Each NF session consisted of 3 feedback blocks with a duration of 8 minutes separated by 5 minutes rest. However, decreases of AA were observed only during the first 5 minutes in blocks 1 and 2, and the first 7 minutes of the last block. The pattern did not change over the course of the NF sessions indicating that our depressed participants did not improve their ability to influence AA over time even when their clinical condition improved. Future studies will have to examine whether the optimal duration of NF blocks is less than the 8 minute blocks used in our study or that longer periods of rest between blocks increase this optimal duration.

It should be noted that the largest clinical effects were observed in the female participants. Earlier studies did not address the effect of gender in the effectiveness of NF for MDD, although in the study by Choi et al [23] 8 out of 10 participants were female. This gender difference may be explained by several reasons. First, the female participants entered the study with significantly less severe depressive symptoms. Given abundant evidence that lower baseline levels of depression severity are associated with better therapeutic outcome (e.g., [29], their less severe symptomatology will have made it more likely that female participants would meet criteria for clinical remission. Second, recent work has indicated that in females MDD is stronger linked to relatively less left- than right frontal activity than men, which makes them potentially better candidates for a treatment that aims at a reduction of AA [12], [15]. This notion is indirectly supported by the finding that only in women treatment response to antidepressants appears related to the presence of AA [18], [19].

Our conclusions are limited for several reasons. First, this was a small, open pilot study. The clinical effects may be confounded by non-specific effects of the procedure (e.g., contact with investigators, behavioural activation as a result of participation) and/or a beneficial natural course. To examine whether this NF treatment is efficacious in MDD, a randomized controlled trial is necessary. This trial should include a credible sham-NF arm to which participants and investigators are blind. If proven efficacious, it is highly unlikely that all depressed patients will respond equally well to NF. Future work will have to address which patients profit most from an intervention aimed at reducing their AA.

## Supporting Information

Protocol S1 **Study protocol.**
(DOCX)

Checklist S1 **TREND Statement Checklist.**
(PDF)
