# Supplementary material for: Neurofeedback As a Treatment for Major Depressive Disorder – A Pilot Study
Source: PLoS One. 2014 Mar 18;9(3):e91837. doi: 10.1371/journal.pone.0091837 (PMC3958393; doi:10.1371/journal.pone.0091837)
Supplement: Protocol S1 — Study protocol. (DOCX) [file pone.0091837.s001.docx]

**Pilot- study into the effectiveness of neurofeedback as a treatment for major depressive disorder**

F.P.M.L. Peeters, MD, PhD

R. Lousberg, PhD

Correspondence:

Dr. F.P.M.L. Peeters

Afdeling Psychiatrie

Academisch Ziekenhuis Maastricht

Postbus 5800

6202 AZ Maastricht

Tel      : 043-3874130

Fax     : 043-3875444

E-mail: [f.peeters@maastrichtuniversity.nl](mailto:f.peeters@maastrichtuniversity.nl)

**Introduction:**

Evidence-based treatments for major depressive disorder (MDD) possess moderate efficacy. However, applicability, availability and acceptability of these treatments are limited. Antidepressant treatment is not acceptable for a vast number of patients with MDD, resulting in high rates of non-compliance. Short-term psychotherapy is not effective in the majority of these patients and not routinely available. Moreover, It is estimated that at least 10-20% of all patients do not respond at all to these treatments even when applied simultaneously and in residential contexts. Therefore, new, acceptable, and efficacious treatments for MDD will help to fill the therapeutic gap that is left by current evidence-based treatments.

**Neurofeedback**

There is a growing interest in neurofeedback (NF) as a treatment for psychiatric disorders including MDD {Lofthouse, 2011 #4632}. It is thought that this technique, within an operant conditioning framework, helps individuals to regulate cortical elektroencephalographic (EEG) activity while receiving feedback from a visual or acoustic signal. The resulting change in EEG activity is presumed to be associated with a change in underlying cortical activation, and subsequently to result in a reduction of associated psychiatric symptoms {Evans, 1999 #2911}. NF is an outstanding example of a non-invasive brain-computer interface which may be particularly appealing to younger patients. Despite widespread claims of efficacy of NF in MDD, the scientific literature consists only of some case-reports and 1 case-control study.

Nevertheless, NF may be an efficacious treatment for MDD.

**Neurophysiology of depression**

Results from fundamental research indicate that MDD appears to be characterized by relatively more left than right resting (alpha, 8-13 Hz) activity in prefrontal regions {Stewart, 2011 #4491}, although some inconclusive studies exist (e.g., {Pizzagalli, 2002 #4526}. This finding has become known as alpha-asymmetry (AA) in MDD. AA is considered as a representation of reduced approach-related behaviours and reduced sensitivity to rewards in MDD {Stewart, 2010 #4493}.

**Neurofeedback als a treatment for MDD**

The intervention will consist of reducing AA in the prefrontal cortex with the use of NF. Participants will learn to increase elektrophysiological brain activity in the left prefrontal cortex in comparison to activity in their right prefrontal cortex. NF-sessions will take place in a maximum of 30 sessions three times each week. Every session will have a duration of 3x8 minutes separated by 4 minutes rest. Primary goal of the pilot-study is obtaining preliminary evidence about possible effectiviness of NF as a treatment for MDD. If at least 50% of the participants will respond (>50% reduction of symptomatology) at the end of treatment, this will considered as an indication of possible effectiveness. Further research in the context of a RCT will be carried out to establish efficacy.

**Hypothesis**

In this study, we have the following research objective:

1. NF aimed at reduction of AA is associated with a reduction in depressive symptomatology.

**Method**

*Design:*

10 Participants, meeting criteria for MMD according to DSM-IV-TR, will be recruited at the unit for treatment of mood disorders at the RIAGG Maastricht, and by advertisements in local media. Formal axis-1 diagnosis will be established with the SCID-1, a formal classification instrument for DSM-IV axis-1 disorders {First, 1995 #4782}. After signing informed consent, subjects will participate in the study.

*Inclusion criteria:*

- Primary axis-1 disorder of Major Depressive Disorder fulfilling DSM-IV criteria {APA, 1994 #87}.
- Written informed consent.

*Exclusion criteria:*

- History of brain trauma (commotio or constusio cerebri)
- Current use of antipsychotics, moodstabilizers or benzodiazepines. Current use of antidepressants is permitted if this medication is not changed within a period of 6 weeks prior to participation in the study. Additionally, no changes in antidepressant medication are allowed during active participation in the study.
- Chronic depression (> two years duration).
- Dysthymia as a primary axis-1 diagnosis
- Bipolar disorder as a primary axis-1 diagnosis
- Severe suicidality (HDRS item # 3 with a score >2) or severe depression symptomatology (HDRS score > 25).
- Pregnancy.
- Comorbid anxiety disorders and personality disorders are permitted.

*Outcome measures*

Main outcome measures are the Quick Inventory of Depressive Symptoms (QIDS-SR; {Rush, 2003 #2772}, the Hamilton Depression Rating Scale (HDRS; {Hamilton, 1967 #1598}, the Remission from Depression Questionnaire (RDQ; {Zimmerman, 2013 #5538} and change in AA between frontal cortical regions.

- The QIDS-SR is a 16-item self-rating scale to assess symptom severity of MDD and will be administered prior to each NF session (3 times a week).
- The HDRS is a 17-item clinician-rated scale for estimating severity of depression during the past week. It will be administered once a week by a trained research assistant during the 6-week study period.
- Before and after every NF session, participants will fill out the PANAS and the TEPS. The PANAS measures momentary positive and negative affects, the TEPS measures anhedonia. Goal of obtaining these instruments is to assess changes in emotions after each NF session.
- At baseline, demografic and medical information will be collected.

*Intervention:*

After informed consent is obtained, the NF intervention will consist of maximum 30 sessions, each lasting 3 x 8 minutes divided by 2 breaks of 4 minutes. Prior to and after each session, baseline EEG alpha-activity will be measured at right- and left frontal regions. Sessions will be done 3 times a week. If after 30 sessions no improvement in clinical condition has occurred, we will offer participants an evidence-based treatment in our outpatient mental health center (RIAGG Maastricht) or the outpatient facility of the department of psychiatry at the Maastricht University Hospital. Additionally, the NF treatment will be stopped in cases of severe suicidality or severe depression symptomatology ( QIDS-SR score > 21). In these cases, the research assistant will contact the principal investigators who will responsible for referral for standard depression treatment.

**Procedure**

After signing informed consent, participants will be invited to the neurofeedback laboratory of the school for Mental Health and Neuroscience (MheNS). This laboratory is a facility that meets the requirements as outlined in NEN 60601 by the Nederlands Normalisatie-instituut. The intervention will take place while participants sit behind a table and look at a monitor on top of this table. The monitor displays a visual feedback signal (thermometer) based on real-time analysis of their elektrophysiologidal data. The participants are instructed to raise the bar of the thermometer (which is indicative of a decrease in AA). During the NF sessions, while being in the laboratory, participants are in constant contact with the research assistant through an audio-channel, additionally there is constant video-surveillance. Prior to the NF session, depression-severity will be assessed as outlined above (see outcome measurements).

EEG-elektrodes will be attached following the international 10-20 system at F3, F4, C3, C4, P3 en P4. EEG will be referenced by 2 elektrodes attached to the earlobes. Under and above the left eye an elektrode will be placed to measure EOG. The elektrode will be applied after cleaning the skin with scrubgel. The impedance on all locations will be kept lower than 5 KΩ.

At the start of each NF session, the baseline EEG is being measures without NF to assess baseline AA which serves as a starting point for feedback. Measuring baseline prior to each session is necessary as can be expected that, at least in the active arm, AA decreases over time. Data collection will be channeled through an acquisition PC with a BrainAmp DC EEG amplifier (Brain Products) using a 1000 Hz sample frequency. Online calculations are done by a filter written for BrainVision RecView. The data will be epoched online into 2.048-s epochs that overlap by 75% and then transformed by a fast Fourier transform (FFT) to the frequency domain (frequency resolution 0.488 Hz). Every 0.512 second, the power within the alpha frequency band (7.8 Hz – 13.1 Hz) of both F3 and F4 will be calculated. AA is computed as the difference of the natural log-transformed F3 and F4-alpha power: Ln(F3-alpha) – Ln(F4-Alpha). Current asymmetry is subsequently compared to the personal mean baseline asymmetry. The result of the calculation will be sent to a stimulus PC running Presentation stimulus delivery software (Neurobehavioral Systems) with an 8-bit parallel port (LPT-port) to control a paradigm showing a visual representation of the asymmetry. In the Presentation paradigm, the last 20 values of the asymmetry are used in a moving average to prevent ‘jitter’ in the feedback. Participants receive feedback with visual feedback; they are instructed to increase the level of a thermometer that is shown on a flatscreen. Additionally, a numerical score below the thermometer indicates their actual total performance. This score is adjusted (i.e. increased) continuously by a number ranging from 0 and 128, depending on the level of the thermometer. In this way a good actual performance (a shift in asymmetry in the desired direction) results in an increasing total score. A big shift in the desired direction results in a rapidly increasing total score, whereas a small shift in the desired direction results in a slow increasing total score. A shift in the undesired direction produced no change in total score. The purpose of this total performance score is to give participants feedback on the differential effect of the sessions.

# **Ethical issues**

Participants will be receive written and verbal information about the procedure and have the right to discontinue participation at any moment. Partcipants wil receive either an experimental or sham experimental treatment for their depressive disorder. To minimize burden resulting from participation in the study, the duration of the study is kept to a maximum of 6 weeks. This duration is based on data from our pilot-study and is totally comparable to the duration of RCT’s that examine novel antidepressants with the use of placebo. Given the extensive waiting-lists for regular evidence-based treatments for depression (3-4 months), participation in the study does not lead to additional delay in the start of standard therapy. Thus, those participants that wish to receive standard antidepressant treatment after participation in the study, will receive such treatment within a normal time-frame.

As mentioned earlier, no side-effects of NF are reported in the literature. Moreover, in our pilot-study in depressed participants, we did not observe any side-effects.

There will be an independent physician available to address all questions and concerns of potential participants. All participants will provide written informed consent. Their data will be collected in a anonymized in a central database in our laboratory. Participants can withdraw consent at any moment without any restrictions. If requested, all participants can be informed of the final results after completion of the study.

All data will be coded and stored anonymously. Each participant will be assigned to an unique code. Access to data and code are restricted to research assistants and principal investigators. The code that entails information about active or sham-treatment will be opened after completion of the study.

**Reimbursment**

All participants will have traveling expenses reimbursed.

**Legal issues**

A WMO- insurance will be taken out for all participants.

# **Publication of results**

After analyses, the study data will be anonymously presented on scientific conferences and published in scientific journals.

**Independent physician**

Dr. K. Schruers, psychiater Mondriaan Zorggroep, locatie Vijverdal.

Vijverdalseweg 1

6226 NB Maastricht

043-3685444
